# Developmental changes in neural letter‐selectivity: A 1‐year follow‐up of beginning readers

**DOI:** 10.1111/desc.12999

**Published:** 2020-06-10

**Authors:** Alice van de Walle de Ghelcke, Bruno Rossion, Christine Schiltz, Aliette Lochy

**Affiliations:** ^1^ Psychological Sciences Research Institute and Institute of Neuroscience Université Catholique de Louvain Louvain‐la‐Neuve Belgium; ^2^ CNRS‐CRAN Université de Lorraine Nancy France; ^3^ Service de Neurologie CHRU‐Nancy Université de Lorraine Nancy France; ^4^ Department of Behavioral and Cognitive Sciences Institute of Cognitive Science and Assessment Université du Luxembourg Esch‐sur‐Alzette Luxembourg

**Keywords:** 1‐year follow‐up, beginning readers, developmental changes, FPVS‐EEG, letter selectivity, reading neural circuits

## Abstract

The developmental course of neural tuning to visual letter strings is unclear. Here we tested 39 children longitudinally, at the beginning of grade 1 (6.45 ± 0.33 years old) and 1 year after, with fast periodic visual stimulation in electroencephalography to assess the evolution of selective neural responses to letter strings and their relationship with emerging reading abilities. At both grades, frequency‐tagged letter strings were discriminated from pseudofont strings (i.e. letter‐selectivity) over the left occipito‐temporal cortex, with effects observed at the individual level in 62% of children. However, visual words were not discriminated from pseudowords (lexical access) at either grade. Following 1 year of schooling, letter‐selective responses showed a specific increase in amplitude, a more complex pattern of harmonics, and were located more anteriorly over the left occipito‐temporal cortex. Remarkably, at both grades, neural responses were highly significant at the individual level and correlated with individual reading scores. The amplitude increase in letter‐selective responses between grades was not found for discrimination responses of familiar keyboard symbols from pseudosymbols, and was not related to a general increase in visual stimulation responses. These findings demonstrate a rapid onset of left hemispheric letter selectivity, with 1 year of reading instruction resulting in increased emerging reading abilities and a clear quantitative and qualitative evolution within left hemispheric neural circuits for reading.

## Graphical abstract

A group of 39 young children were tested with FPVS‐EEG to understand changes in the left hemisphere when learning to read. After only 1 year of schooling, a quantitative and qualitative evolution in the selective neural responses to letters is found, independently of a general increase of visual stimulation responses.

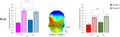

**Research highlights**

- Left lateralized hemispheric letter selectivity is triggered by emerging reading abilities. After 1 year of schooling, it evolves quantitatively and qualitatively independently of lexical access.
- The quantitative evolution in category‐selectivity is not related to a general increase of visual stimulation responses, and is not observed for familiar keyboard symbols.
- Letter‐selective neural responses are highly significant at the individual level and correlate with individual reading scores.
- The FPVS‐EEG approach presents high potential for early detection of reading acquisition disorders.

## 1 INTRODUCTION

Reading is a complex brain function, which lies at the interface of several cognitive domains including vision and language. Adults’ reading performance is impressive: an average speed of 200 ms per word (Rayner, 1998) encompasses the automatic and mandatory identification of each letter in a string, the analysis of their order, and the access to the meaning of the word (e.g. Stroop effect, Glaser & Glaser, 1989 and priming studies, Van Orden, Johnston, & Hale, 1988). However, reaching this level of expertise requires a slow and tedious acquisition process during development: children start by learning the correspondence between single letters and sounds, then combinations of letters and small words, requiring years of instruction before a fluent adult‐like level is attained (Rayner, Foorman, Perfetti, Pesetsky, & Seidenberg, 2001). This expertise is difficult to acquire for a significant proportion of children and adults, who have difficulties ranging from poor reading abilities to specific reading disorders (Kutner et al., 2007; Peterson & Pennington, 2015).

In adults, reading relies on a left hemispheric (LH, hereafter) specialized brain network, in particular in the ventral occipito‐temporal cortex (VOTC) (Carreiras, Armstrong, Perea, & Frost, 2014; Dehaene, Cohen, Sigman, & Vinckier, 2005; Lochy et al., 2018; Wandell, 2011). The LH specialization for reading is thought to emerge with children's acquisition of grapheme‐phoneme (GP, hereafter) mappings, which either induces the establishment of connections (Phonological Mapping Hypothesis, Maurer & McCandliss, 2007), or relies on pre‐existing connections (Saygin et al., 2016; Stevens, Kravitz, Peng, Tessler, & Martin, 2017) between anterior language areas and posterior visual regions. By capturing automatic processes without necessarily requiring explicit reading, classical electroencephalographic (EEG) studies have distinguished between ‘coarse‐grained tuning’, in which a different response is observed for real letter strings than for non‐letter objects (letter sensitivity; e.g. faces, shoes) or letter‐like stimuli (letter selectivity; e.g. pseudofonts, symbols, or digits), and “fine‐grained tuning” (or lexical access), in which a different response is observed for words than for non‐legal letter strings (e.g. consonants, non‐words, or pseudowords), arising from sensitivity to well‐formed patterns of assembled letters (Centanni, King, Eddy, Whitfield‐Gabrieli, & Gabrieli, 2017; Centanni et al., 2018; Coch & Meade, 2016; Eberhard‐Moscicka, Jost, Raith, & Maurer, 2015).

However, the developmental course of this LH specialization for reading remains unclear: *when* does it emerge and *how* does it evolve during reading acquisition? One of the reasons for this uncertainty is the difficulty of differentiating neural changes during the first years of schooling that are due to general age‐related changes (e.g. sensory, attentional, or brain maturation changes) or to specific improvements in reading ability. Another reason is the lack of sensitivity of individual reading ability measures, even though such measures are critical for understanding typical and deviant reading acquisition mechanisms (e.g. Wandell, Rauschecker, & Yeatman, 2012).

Initial longitudinal EEG studies suggested that the left lateralized hemispheric letter sensitivity/selectivity is a slow process requiring the automatization of GP mappings ability, which takes several years (Eberhard‐Moscicka et al., 2015; Maurer, Brem, Bucher, & Brandeis, 2005; Maurer et al., 2006). However, more recent studies have evidenced left lateralized letter selectivity even before formal instruction in 5‐year‐old preschool children (with frequency‐tagging EEG: Lochy, Van Reybroeck, & Rossion, 2016; with functional magnetic resonance imaging (fMRI): Dehaene‐Lambertz, Monzalvo, & Dehaene, 2018). While differences between visual letters and objects have already been shown in 4‐year‐old children (Cantlon, Pinel, Dehaene, & Pelphrey, 2011), letter selectivity was reported only recently in fMRI at the visual word form area (VWFA) in 5‐ and 6‐year‐old children (Centanni et al., 2018), using single letters rather than letter strings. Concerning lexical access, there is agreement from both longitudinal and transversal EEG studies that it emerges later, at about the fourth year of elementary school (Coch & Meade, 2016), and therefore is not related to letter sensitivity or to early reading abilities at the behavioral level (Eberhard‐Moscicka et al., 2015; Zhao et al., 2014).

Concerning the issue of *how* letter sensitivity/selectivity evolves, a recent longitudinal fMRI study, with recording sessions 2 month‐apart from the end of preschool to the beginning of grade 2, showed that the volume of brain tissue activated by letter strings followed an inverted U‐curve, with an initial increase and then a later decrease in the amount of responding voxels (Dehaene‐Lambertz et al., 2018). Yet, at the peak of the VWFA, the activation increased steadily along the different testing sessions. In a large‐scale longitudinal EEG study, an inverted U‐curve development has also been shown: after an initial increase, the assessment from second to fifth grade revealed a decrease in the amplitude of the N1 event‐related potential component associated with letter sensitivity/selectivity (Maurer et al., 2011) and this decrease was also found when comparing second graders to adults (Maurer et al., 2006). This non‐linear development was interpreted as an effect of reading practice: an initial high sensitivity for visual aspects of print is followed by a more selective sensitivity arising with reading acquisition (Brem et al., 2010; Maurer et al., 2005, 2006, 2011). In contrast, other fMRI studies instead suggested an increase in amplitude of signal responses in the VOTC with age and behavioral improvement in reading ability (Ben‐Shachar, Dougherty, Deutsch, & Wandell, 2011; Booth et al., 2001; Centanni et al., 2017; Turkeltaub, Flowers, Lyon, & Eden, 2008). A different proposal emerged from the finding in preschool children of a right‐lateralized N1 modulated by letter knowledge: an early right hemispheric (RH) letter tuning that reflects only visual familiarity with letter shapes would precede the emergence of a LH letter tuning due to visual‐to‐phonological associations (Brem et al., 2013; Maurer et al., 2005, 2007).

Besides signal strength (i.e. amplitude), the scalp topography of responses also seems to change with development. Olulade, Flowers, Napoliello, and Eden (2013) showed that letter selectivity in children (10.2 ± 3.0 years old) was left lateralized but located at a more posterior site than in adults. In recent EEG frequency‐tagging studies, letter selective responses were recorded quasi‐exclusively over a posterior left electrode (O1 of the 10–20 electrode system) in 5‐year‐old preschool children (Lochy et al., 2016), while in adults the electrode capturing the highest response was more lateral over the LH (Lochy, Van Belle, & Rossion, 2015).

Discrepancy in findings across studies might be partly due to methodological differences and individual variability, which is high at those ages. Indeed, differences observed in group grand‐averaged waveforms may be absent in individual results, due to a lack of measure sensitivity, a critical aspect of developmental studies of reading. Also, there are further sources of variability in the selection of electrodes, time windows, and quantification methods that hamper the reliability and reproducibility of results (Thigpen, Kappenman, & Keil, 2017).

In the present study, we aimed at investigating the developmental course of the LH specialization for reading. Our main objective was to assess the quantitative and qualitative evolution of selective neural responses to letter strings and their relationship with emerging reading abilities. To do this, we tested a large group of children (*N* = 39) at the beginning of formal reading instruction and 1 year later, both behaviorally and with frequency‐tagging, also known as fast periodic vsual stimulation, combined with EEG (FPVS‐EEG). This approach is particularly suitable to measure automatic discrimination of a categorical change: for instance, when streams of non‐words are presented at 6 Hz and words are inserted periodically every five items, thus at 1.2 Hz. If words are discriminated from non‐words, it gives rise in the EEG frequency domain to a peak of response amplitude at 1.2 Hz and its harmonics (i.e. exact integers of 1.2: 2.4, 3.6 Hz, etc.) (for a review: Norcia, Appelbaum, Ales, Cottereau, & Rossion, 2015). This measure of a differential processing between two categories of stimuli has shown high sensitivity at the individual level (for letter strings: Lochy et al., 2015; for visual quantities or faces: Guillaume, Mejias, Rossion, Dzhelyova, & Schiltz, 2018; Liu‐Shuang, Norcia, & Rossion, 2014), is highly objective (i.e. responses are extracted exactly at frequencies pre‐defined by the experimenters), and demonstrates high test‐retest reliability (Dzhelyova et al., 2019), thus being well‐suited for a longitudinal study.

Different levels of automatic discrimination were tested here. First, to test letter selectivity, words or pseudowords (W and PW respectively) were presented among pseudofont strings (PF‐W and PF‐PW conditions). Second, to test lexical access, words were presented among pseudowords (PW‐W condition). Third, non‐specific/general age‐related changes were tested by measuring responses to familiar keyboard symbol strings among ‘pseudosymbol’ strings (PSY‐SY condition). This condition also allowed a control for low‐level visual processes related to strings of printed characters that have similar characteristics than letters (assembled features with curves, junctions, high spatial frequency, etc).

In grade 1, we expected a left lateralized letter selectivity as previously found in 5‐year‐old preschool children with the same approach (Lochy et al., 2016), but did not yet expect lexical responses. We also expected a positive relationship with reading abilities, at both grades (Centanni et al., 2018; Lochy et al., 2016). Following 1 year of schooling, we expected an increase in letter selectivity, together with the improvement of reading performances and the potential emergence of lexical responses. The expected increase in letter selectivity may reflect a refinement of neural tuning for letters, yet it could manifest in different ways, both quantitatively and qualitatively. At the quantitative level, the amplitude of the response on the scalp should be higher after 1 year of experience, given previous observations in the same age‐range showing increases in signal strength for letter sensitivity (Ben‐Shachar et al., 2011; Booth et al., 2001; Dehaene‐Lambertz et al., 2018; Maurer et al., 2006, 2011; Turkeltaub et al., 2008). At the qualitative level, the scalp topography of the response could become more anterior, as suggested by previous findings comparing children and adults (Lochy et al., 2015, 2016; Olulade et al., 2013). In addition, we also tested whether the pattern of harmonic frequency responses changed with development. Based on the same approach in the domain of face perception development (Lochy, de Heering, & Rossion, 2019), we expected that the response would be concentrated on the first harmonic in grade 1, and more distributed to higher harmonics in grade 2.

## 2 MATERIAL AND METHODS

### 2.1 Participants

Forty‐one children from two different French‐speaking Belgian schools were tested twice 1 year apart. Testing took place during the first trimester of grade 1 (i.e. after 2–3 months of formal reading instruction; mean age = 6 years, 5 months; range = 5 years, 11 months ‐ 7 years, 2 months; 18 boys, 38 right‐handed) and 1 year after (two children were excluded because of abnormal performances in behavioral tests; see below). All children had normal or corrected‐to‐normal vision, and were unaware of the goal of the study. The parents gave written, informed consent for the study, which was approved by the Biomedical Ethical Committee of the Université Catholique de Louvain. The testing took place in a quiet room of the school in two or more sessions (EEG, behavioral).

### 2.2 Behavioral testing

In grade 1, general cognitive functions and reading ability were assessed by means of standardized tests: nonverbal intelligence (CPM; Raven, 1998), selective attention (TEA‐Ch; Manly, Robertson, Anderson, & Mimmo‐Smith, 2004), vocabulary production (N‐EEL; Chevrie‐Muller & Plaza, 2001), and reading of single letters, syllables, pseudowords, regular and irregular words (BELO; George & Pech‐Georgel, 2012, BALE; Jacquier‐Roux, Lequette, Pouget, Valdois, & Zorman, 2010) (Table 1). To identify outliers within the distribution of the sample, individual *Z* scores were computed for each general cognitive function. One child was excluded because of scores lower than 2 standard deviations (*SD*) in all general cognitive functions. Another child was excluded because of a medicated attentional disorder. In grade 2, we only reassessed reading ability by means of the same standardized subtests used in grade 1, except for regular and irregular words, that were assessed with 12 items (per category) of one standardized battery in grade 1 (BELO, George & Pech‐Georgel, 2012) and with 20 different items (per category) in grade 2 (BALE, Jacquier‐Roux et al., 2010). However, the items were matched across grades in standard frequency index (all *p* values >.050) as well as in number of letters (all *p* values >.050).

**TABLE 1:** Descriptive statistics of behavioral testing scores (*N* = 39)

Behavioral tests and sub‐tests	Grade 1			Grade 2		
	Min	Max	Mean (*SD*)	Min	Max	Mean (*SD*)
General cognitive functions						
Nonverbal intelligence/36 (CPM, accuracy in %)	55.56	97.22	77.71 (11.39)	—	—	—
Selective attention (TEA‐Ch, speed in sec)	2.35	11.74	6.37 (1.92)	—	—	—
Vocabulary production/114 (N‐EEL, accuracy in %)	58.77	94.74	78.72 (8.92)	—	—	—
Reading ability						
Single letters/26 (BELO, accuracy in %)	30.77	100.00	74.16 (18.02)	53.85	100.00	88.16 (10.67)
Syllables/74 (BELO, accuracy in %)	0.76	80.00	43.36 (20.96)	4.54	91.51	68.73 (21.10)
Pseudowords/20 (BALE, accuracy in %)	0.00	70.00	16.41 (17.73)	0.00	90.00	50.51 (25.75)
Regular words/12 (BELO, accuracy in %)	0.00	91.67	19.66 (23.76)	—	—	—
Irregular words/12 (BELO, accuracy in %)	0.00	50.00	7.91 (12.23)	—	—	—
Regular words/20 (BALE, accuracy in %)	—	—	—	0.00	100.00	63.33 (27.89)
Irregular words/20 (BALE, accuracy in %)	—	—	—	0.00	85.00	33.46 (23.14)
Composite score/184 (BELO, BALE, accuracy in %)	4.22	74.87	36.45 (2.87)	9.00	85.37	63.80 (3.17)

As it is expected at these stages of formal instruction, descriptive statistics of reading scores highlight only limited (or emergent) reading abilities in each grade, with performances that increase from grade 1 to grade 2 (*t*‐tests, all *P* values <.050; see Table 1). Given the different levels of complexity and types of processes (analytical vs. direct decoding) involved in each reading subtest, we averaged all reading scores in a reliable composite measure of reading, for each child and in each grade. Because the items used for regular and irregular word reading differed across grades, we ran all the analyses with or without this subtest, and the results were similar. In the manuscript, we provide the full composite score, and in Supporting Information, the composite score that does not include word reading.

### 2.3 EEG testing

#### 2.3.1 Stimuli

**FIGURE 1:**
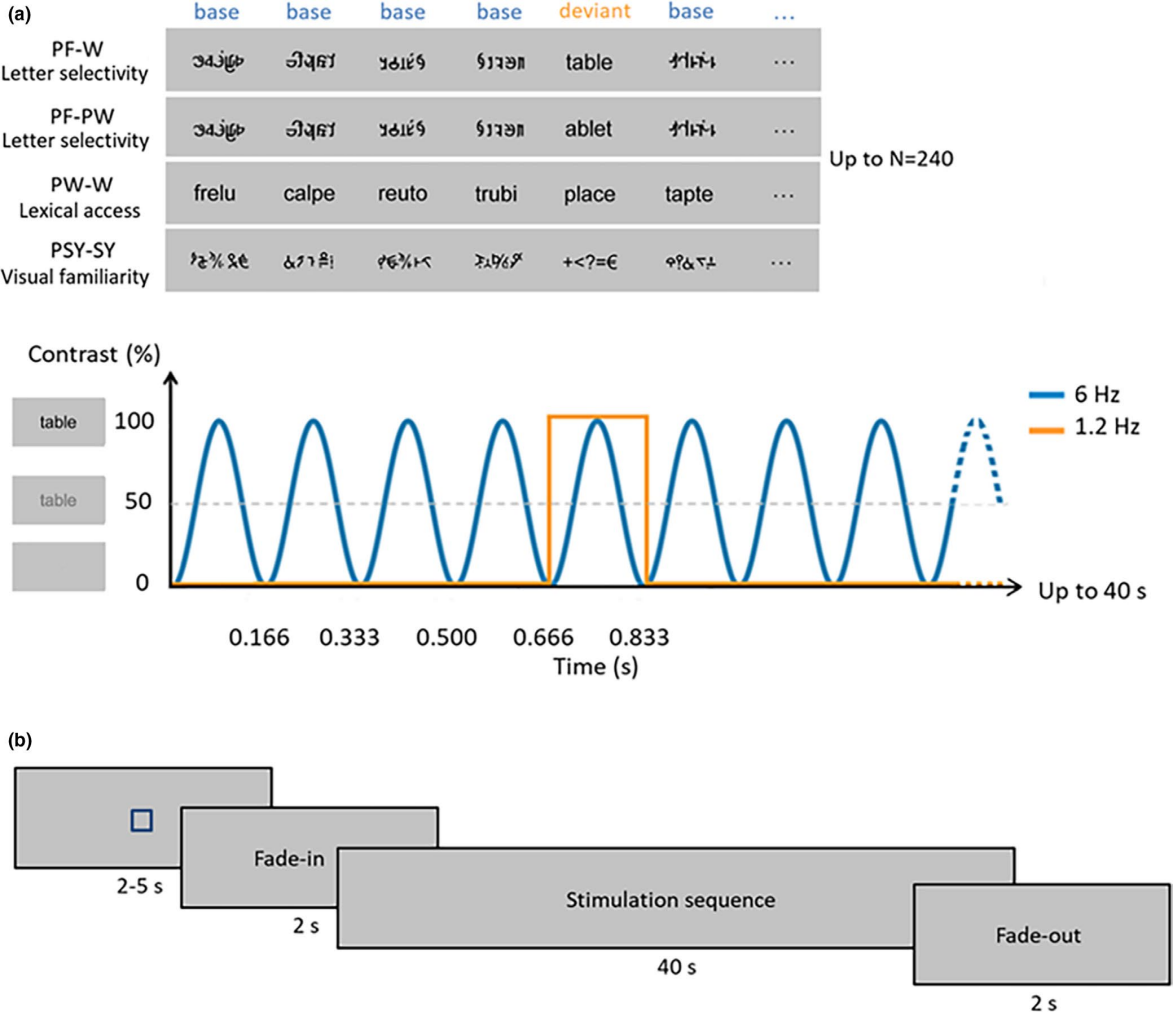
Experimental paradigm. (a) Each stimulation sequence (first, second, third and fourth rows) lasted 40 s, during which stimuli were presented by sinusoidal contrast modulation at 6 Hz, each stimulus reaching full contrast after 83 ms (i.e. one cycle duration = 166.66 ms). Stimulation consisted of four base (B) stimuli followed by one deviant (D) stimulus, i.e. with a pattern of BBBBDBBBBDBBB, etc. Deviant stimuli therefore appeared at 6 Hz/5, so at 1.2 Hz. Stimuli were randomly presented with no immediate repetition and appeared continuously on the screen. In total, 240 stimuli were presented per sequence (48 deviant stimuli and 192 base stimuli), and each condition was repeated three times. The first two conditions (PF‐W, PF‐PW) assessed letter selectivity with deviant words (W; first row) or pseudowords (PW; second row) inserted in base pseudofont strings (PF). The third condition (PW‐W; third row) assessed lexical access with deviant words (W) inserted in base pseudowords (PW). The control condition (PSY‐SY; fourth row) assessed the effect of visual familiarity with deviant symbol strings (SY) inserted in base pseudosymbol strings (PSY). This condition also allowed to control for non‐specific/general age‐related changes. (b) Timeline of a sequence: each sequence started with a fixation square (for 2–5 s) after which the stimulation faded in (for 2 s) then reached full contrast (for 40 s) and then faded out (for 2 s)

Base and deviant stimuli were combined in order to build four different conditions (Figure 1). Two conditions assessed letter selectivity, with deviant letter strings (words or pseudowords, *N* = 20 each) inserted in base pseudofont strings (PF, *N* = 20). The third condition assessed lexical access, with deviant words (W, *N* = 20) inserted in base pseudowords (PW, *N* = 20). The last condition, which aimed at controlling for visual familiarity and for non‐specific/general age‐related changes, assessed discrimination of familiar keyboard symbol strings (SY, *N* = 20) inserted in base pseudosymbol strings (PSY, *N* = 20). Words were selected from the Manulex database (Lété, Sprenger‐Charolles, & Colé, 2004) and were constituted of four (*N* = 10) or five letters (*N* = 10). Pseudowords were pronounceable letter strings respecting the phonological rules in French. They were built one by one on the basis of the words, by changing the position of their constitutive letters (e.g. the words ‘page’ and ‘table’ give rise to the pseudowords ‘gape’ and ‘ablet’). Pseudowords and words were matched in bigram frequency (t(38)=0.20; *p* = .841; PW = 8,141.15 ± 3,491.40 *SD*, W = 8,390.10 ± 4,261.80 *SD*), letter identities, and in letter numbers(four or five). Keyboard symbols (&, €, !, %, ?, =, +, <,>, *) were arranged into four or five element strings. Pseudofont stimuli were built one by one on the basis of the words: each word was vertically flipped and its letters were segmented into simple features using Adobe Photoshop. These segments were then rearranged to form pseudoletters, respecting the total number of characters (four or five) and the overall size (width × height) of the original word (Lochy et al., 2015, 2016). Pseudoletters thus contained junctions, ascending/descending features and close‐up shapes, as real letters. Pseudosymbol stimuli were built similarly on the basis of the symbols. Therefore, each deviant stimulus (W, PW, SY) had a corresponding visual control base stimulus (pseudofont or pseudosymbol) containing the exact same amount of black‐on‐white contrast, so that they were comparable in terms of low‐level visual properties. In all conditions, stimuli were presented centrally in Verdana font with a height between 47 and 77 pixels and a width between 119 and 199 pixels,. At a viewing distance of 1 m with a screen resolution of 800 × 600 pixels and a refresh rate of 60 Hz, stimuli ranged from 3.11 to 5.20 (width) and 1.32 to 2.18 (height) degrees of visual angle.

#### 2.3.2 Procedure

The stimulation procedure was very similar to previous FPVS‐EEG studies on words recognition (Lochy et al., 2015, 2016). A fixation square was displayed for 2–5 s (randomly jittered between sequences), followed by 2 s fade‐in (progressive increasing modulation depth from 0% maximum contrast level to 100%), the stimulation sequence for 40, and 2 s fade‐out. This fading‐in/out procedure was used to avoid abrupt eye‐movements or blinks at the beginning or near to the end of a sequence. Stimuli were presented by means of sinusoidal contrast modulation at a base frequency rate of 6 Hz (i.e. one item every 166.66 ms, from a grey background to full contrast and back in 166.66 ms; thus, each item reached full contrast at 83 ms). Every fifth stimulus (1/5, frequency of 1.2 Hz, thus, every 833 ms), a letter string (PF‐W, PF‐PW and PW‐W conditions) or a symbol string (PSY‐SY condition) was presented (Figure 1). Stimuli were presented with Java SE Version 8. Each condition was repeated three times. Considering a total of 40 s (sequence duration) × 3 (repetitions) × 4 (conditions), 8 min of stimulation were presented in total. There was a pause of approximately 30 s between each sequence, which was initiated manually to ensure low‐artifact EEG signals.

During the stimulation, children performed a color‐change detection task on the fixation square (blue to yellow; 200 ms, six changes randomly timed per sequence) (see Video 1—Supporting Information). This orthogonal task was included to maintain both a central gaze position on the screen and a constant level of attention (Lochy et al., 2015). Performance was almost at ceiling in both grades, showing high attention to the stimulation (PF‐W: 97% ± 9.11 *SD* in grade 1 and 100% ± 0.00 *SD* in grade 2; PF‐PW: 94.60% ± 12.46 *SD* in grade 1 and 99.15% ± 5.34 *SD* in grade 2; PW‐W: 98.20% ± 7.64 *SD* in grade 1 and 100% ± 0.00 *SD* in grade 2; PSY‐SY: 98.25% ± 7.54 *SD* in grade 1 and 99.15% ± 5.34 *SD* in grade 2). An ANOVA performed on response times with *Grade* (grade 1, grade 2) and *Condition* (PF‐W, PF‐PW, PW‐W, PSY‐SY) as within‐subjects factors, showed a main effect of *Grade* (*F*_1,36_ = 47.13, *p* = .000, η^2^ = 0.57); response times being faster in grade 2 (607.20 ms ± 60.32 *SD*) than in grade 1 (720.10 ms ± 109.64 *SD*). There were no other main effects or interactions (all Fs < 1).

#### 2.3.3 Acquisition

During EEG recording, children were seated comfortably in a quiet room in the school. EEG signal was acquired at 1,024 Hz using a 32‐electrode Biosemi Active II system (Biosemi, Amsterdam, Netherlands), with standard 10–20 system locations. The magnitude of the offset of all electrodes, referenced to a common mode sense/driven‐right leg loop, was held below 50 mV.

#### 2.3.4 Preprocessing

All EEG analyses were carried out using Letswave 5.c (https://www.letswave.org) and Matlab 2014 (The Mathworks) and followed procedures validated in several studies using letter strings or face and object stimuli (see e.g. Retter & Rossion, 2016). After band‐pass filtering between 0.1 and 100 Hz, EEG data were segmented to include 2 s before and after each sequence, resulting in 44 s segments. Data files were then downsampled to 256 Hz to reduce file size. Artifact‐ridden electrodes were replaced using linear interpolation with neighboring electrodes. All electrodes were re‐referenced to the common average. EEG recordings were then segmented again from stimulation onset until 39.996 s, corresponding exactly to 48 complete 1.2 Hz cycles within stimulation. This is the largest amount of complete cycles of 833 ms at the frequency of deviant stimuli (1.2 Hz) within the 40 s of stimulation period.

#### 2.3.5 Frequency domain analysis

To reduce EEG activity that is not phase‐locked to the stimulus, the three repetitions of each condition were averaged in the time domain for each individual participant. Then, to convert data from the time domain into the frequency domain, a Fast Fourier Transform (FFT) was applied to these averaged time windows and normalized amplitude spectra were extracted for all electrodes. Thanks to the long time‐window (39.996 s), this procedure yields EEG spectra with a high frequency resolution (1/39.996 s = 0.025 Hz), producing a high SNR (Regan, 1989; Rossion, 2014) and allowing unambiguous identification of the response at the exact frequencies of interest (i.e. 6 Hz and its harmonics for the base stimulation rate and 1.2 Hz and its harmonics for the deviant stimulation rate). All of the responses of interest can be concentrated in a discrete frequency bin at the stimulation frequency, that occupies a small fraction of the total EEG bandwidth. In contrast, biological noise is distributed throughout the EEG spectrum, resulting in a SNR in the bin of interest that can be very high (Regan, 1989; Rossion, 2014). To estimate SNR across the EEG spectrum, amplitude at each frequency of interest (bin) was divided by the average amplitude of 20 surrounding bins (10 on each side) (e.g. Liu‐Shuang et al., 2014).

To quantify the responses of interest in microvolts, the average voltage amplitude of the 20 surrounding bins (i.e. the noise) was subtracted from the amplitude at the frequency bin of interest (e.g. Dzhelyova & Rossion, 2014; Retter & Rossion, 2016) (baseline subtracted amplitudes). To assess the group‐level significance of the responses, *Z* scores were computed on the grand averaged amplitude spectrum for each condition (e.g. Liu‐Shuang et al., 2014; Lochy et al., 2015), at one of five contiguous posterio‐lateral channels determined by visual inspection of the scalp topographies (i.e. P7, O1, Oz, O2, P8). *Z* scores larger than 2.58 (*p* < .005, one‐tailed, signal > noise) were considered significant. As in previous studies (Liu‐Shuang et al., 2014; Lochy et al., 2015; Lochy et al., 2016), a rather liberal statistical threshold was used given that there is less harm in including weak harmonic responses (i.e. adding a baseline‐subtracted harmonic response that is not above noise level is like adding a zero) in the total response amplitude than in failing to include genuine responses. To assess the significance of responses at the individual level, the raw amplitude signal in the frequency‐domain was segmented at each of the harmonics (F/5 or 1.2 Hz up to 6F/5 or 7.2 Hz), with 10 bins on each side of the bin‐of‐interest. Then the amplitude values of these harmonic segments were summed for each condition, and participant. Finally, *Z* scores were computed on this summed amplitude response.

An identical number of harmonics was selected across all conditions and electrodes based on the condition in which the highest number of consecutive harmonics was significant on any of the five electrodes, in order to have the same number of harmonics in the compared conditions. Finally, to quantify the periodic response distributed on several harmonics, the baseline subtracted amplitudes of significant harmonics (except the base stimulation frequency for the responses to deviant stimuli) were summed for each child (Retter & Rossion, 2016).

## 3 RESULTS

### 3.1 Base rate responses

#### 3.1.1 Overall responses and electrodes of interest

**FIGURE 2:**
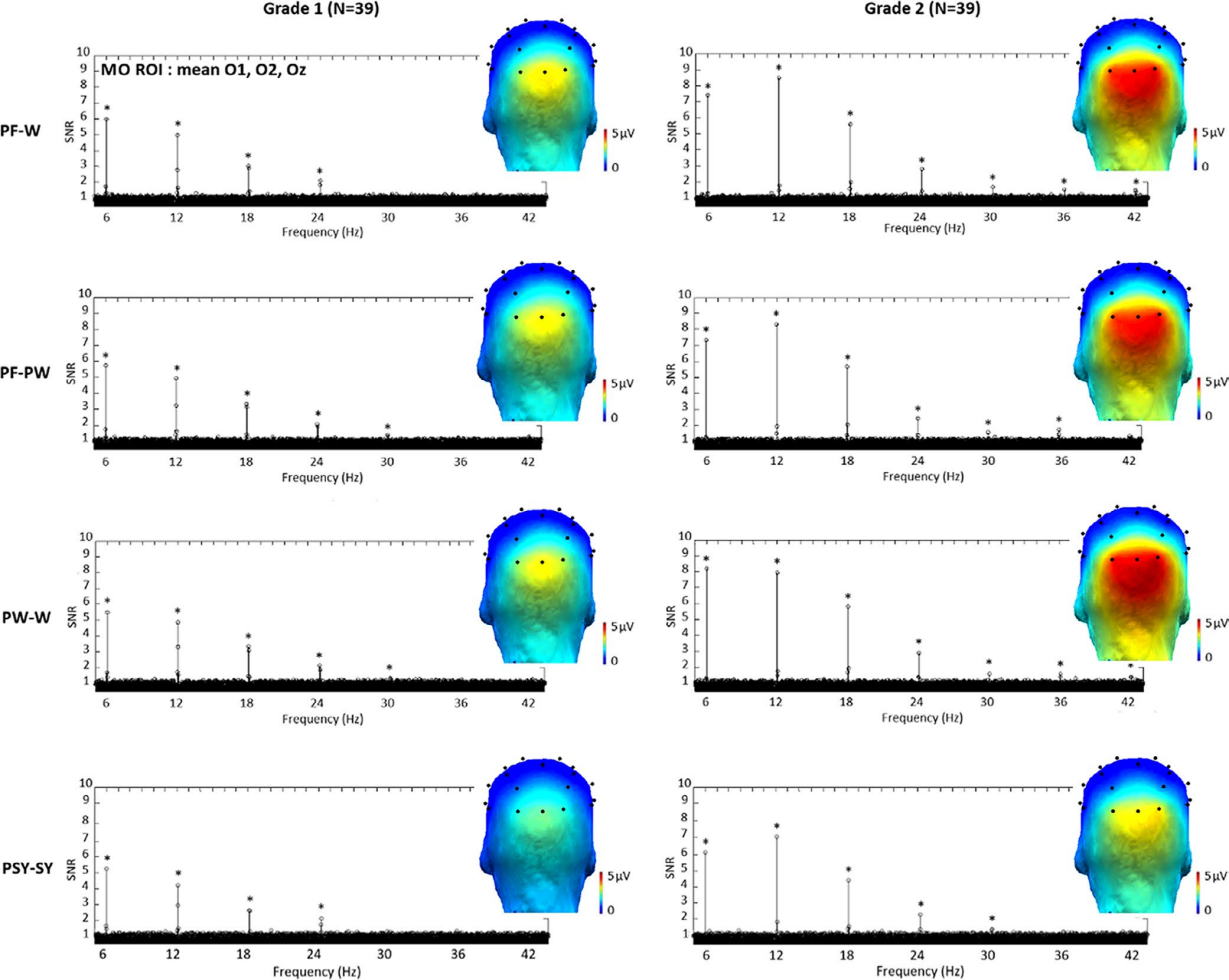
Base rate responses for each condition and grade. Grand‐averaged (*N* = 39) SNR EEG spectra at the MO (middle occipital electrodes) ROI (mean O1, O2, Oz) and scalp topographies (sum of baseline subtracted amplitudes computed on seven significant (*) harmonics from *F* (6 Hz) to 7F (42 Hz); see Methods). In each grade (columns), there were significant responses (*Z* scores > 2.58) in each condition (words or pseudowords within pseudofont strings, words within pseudowords and symbol strings within pseudosymbol strings; PF‐W, PF‐PW, PW‐W and PSY‐SY respectively). In all conditions, response amplitudes were larger in grade 2 than in grade 1

In both grades, significant responses (*Z* score >2.58) were found in all conditions (PF‐W, PF‐PW, PW‐W and PSY‐SY) at exactly 6 Hz and several harmonics at middle occipital (MO) electrodes. As determined by grand‐averaged data (see Methods), the highest number of consecutive significant harmonics were five in grade 1 (from 6 to 30 Hz) and seven in grade 2 (from 6 to 42 Hz) (Figure 2). In order to select an identical number of harmonics across grades, the sum of baseline subtracted amplitudes was computed on the highest number of harmonics (i.e. seven in grade 2). Electrodes were then ranked according to their largest amplitude values. This ranking procedure revealed that in all conditions and in both grades, the largest response was recorded at three MO electrodes: O1 (3.48 µV), O2 (3.73 µV) and Oz (3.65 µV) (see Figure S1—Supporting Information). Based on this, a medial‐occipital ROI was defined for further analysis (MO ROI = mean O1, O2, Oz).

#### 3.1.2 Response pattern and evolution

An ANOVA was performed on the sum of baseline subtracted amplitudes at the MO ROI with *Grade* (grade 1, grade 2) and *Condition* (PF‐W, PF‐PW, PW‐W, PSY‐SY) as within‐subjects factors. It showed a main effect of *Grade* (*F*_1,38_ = 40.48, *p* = .000, η^2^ = 0.52), responses being overall higher in grade 2 (4.34 µV) than in grade 1 (2.91 µV). It also showed a main effect of *Condition* (*F*_3,96_ = 28.77, *p* = .000, η^2^ = 0.043), with the response amplitude of the PSY‐SY condition (2.83 µV) being lower (all *t*‐tests *p* values = .000) than the response amplitude of the PF‐W (3.90 µV), PF‐PW (3.81 µV) and PW‐W (3.94 µV) conditions, which did not differ from each other. The interaction between *Grade* and *Condition* was also significant (*F*_2,83_ = 4.04, *p* = .018, η^2^ = 0.096), the amplitude difference between PSY‐SY and letter strings (mean PF‐W, PF‐PW, PW‐W) conditions was greater (*t*[38] = 4.19; *p* = .000) in grade 2 (1.36 µV) than in grade 1 (0.75 µV) (Figure 2).

### 3.2 Discrimination responses

#### 3.2.1 Overall responses and electrodes of interest

**FIGURE 3:**
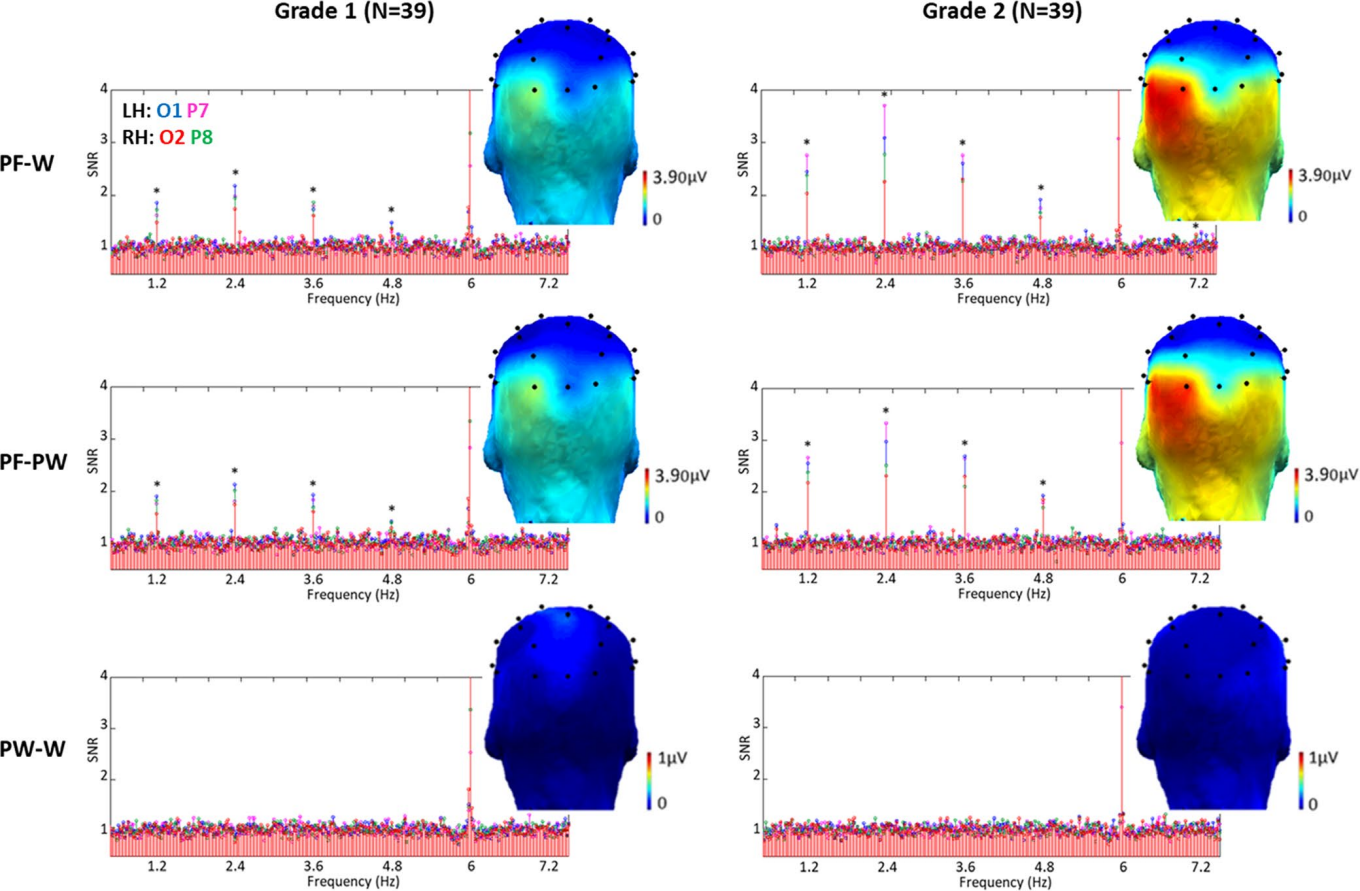
Discrimination responses to letter strings in each condition and grade. Grand‐averaged (*N* = 39) SNR EEG spectra on O1 (blue), P7 (magenta), O2 (red) and P8 (green) and scalp topographies (sum of baseline subtracted amplitudes computed on five significant (*) harmonics excluding the base stimulation frequency; see Methods). In each grade (columns), there were significant discrimination responses (*Z* scores > 2.58) to words or pseudowords within pseudofont strings (PF‐W and PF‐PW conditions) from F/5 or 1.2 Hz to 6F/5 or 7.2 Hz, and no significant discrimination response (*Z* scores < 2.58) to words within pseudowords (PW‐W condition). In PF‐W and PF‐PW conditions, response amplitudes were larger in grade 2 than in grade 1

Discrimination responses were significant (*Z* scores >2.58) in both grades for letter strings (words, pseudowords) and symbol strings within their respective visual control stimuli (PF‐W, PF‐PW and PSY‐SY conditions), at exactly 1.2 Hz and several harmonics at several electrodes. On the contrary, for words within pseudowords (PW‐W condition), there was no significant response (Figure 3).

Significant harmonics were determined on the grand‐averaged data for all participants, and per condition separately. A maximum of five consecutive harmonics (from 1.2 to 7.2 Hz, excluding the base rate at 6 Hz) were found to be significant in grade 2, and four in grade 1 (see Methods). We have thus computed the sum of five harmonics for each condition, in order to have the same number of summed harmonics in the compared conditions (Figure 3). Electrodes were then ranked according to their largest amplitude values, which were all in the LH. In PF‐W and PF‐PW conditions, the largest response was recorded on O1 (PF‐W: 2.23 µV, PF‐PW: 2.28 µV) in grade 1 but on P7 (PF‐W: 4.03 µV, PF‐PW: 3.74 µV) in grade 2, the response then decreasing by more than half on a more dorsal electrode PO3 (grade 1, PF‐W: 0.94 µV and PF‐PW: 1.05 µV; grade 2, PF‐W: 1.39 µV and PF‐PW: 1.41 µV). In PSY‐SY condition, the largest response was recorded on the same electrodes as well as on their right homologous electrodes in each grade (grade 1: O1 = 1.24 µV, P7 = 0.60 µV, O2 = 1.16 µV, P8 = 1.04 µV; grade 2: O1 = 0.82 µV, P7 = 0.76 µV, O2 = 0.78 µV, P8 = 0.93 µV) (see Figure S2—Supporting Information). Based on this ranking procedure, we selected the left O1 and P7 postero‐lateral electrodes and their homologous right postero‐lateral electrodes O2 and P8 for analyses.

#### 3.2.2 Response pattern and evolution for letter strings

Given that the condition assessing lexical access (PW‐W) did not give rise to significant discrimination responses at 1.2 Hz and its higher harmonics (Figure 3), we first tested whether the amplitude values, computed similarly as for the other conditions (see above), contained any signal above noise‐level by performing independent *t*‐tests against zero for each electrode (O1, O2, P7, P8) and grade level (grade 1, grade 2). These four *t*‐tests did not reveal any significant response (all *p* > .13). As it would artificially give rise to an effect of *Condition* and interactions with *Condition*, the PW‐W condition was not included in a main ANOVA. Nevertheless, this analysis is provided as Supporting Information (see Analysis 1).

##### Response amplitudes

An ANOVA was performed on the sum of baseline‐subtracted amplitudes of responses to letter strings with *Grade* (grade 1, grade 2), *Condition* (PF‐W, PF‐PW), *Hemisphere* (LH, RH) and *Electrode Position* (posterior‐O1/O2, lateral‐P7/P8) as within‐subjects factors. There was a significant main effect of *Hemisphere* (*F*_1,38_ = 10.97, *p* = .002, η^2^ = 0.22), responses being larger in the LH (2.89µV) than in the RH (2.22µV), and a main effect of *Grade* (*F*_1,38_ = 58.94, *p* = .000, η^2^ = 0.61), letter‐selective responses being overall larger in grade 2 (3.30 µV) than in grade 1 (1.81 µV). There was also a significant interaction between *Grade* and *Hemisphere* (*F*_1,38_ = 6.31, *p* = .016, η^2^ = 0.14), qualified by a significant triple interaction between *Grade*, *Hemisphere* and *Electrode Position* (*F*_1,38_ = 4.28, *p* = .045, η^2^ = 0.10). There were no other main effects or interactions (all *F*s < 1) (Figure 3).

To decompose the triple interaction between *Grade*, *Hemisphere*, and *Electrode Position*, the next analyses assessed the effects by grade using ANOVAs with *Hemisphere* (LH, RH) and *Electrode Position* (posterior‐O1/O2, lateral‐P7/P8) as within‐subjects factors. Given that no effect of *Condition* or interaction with *Condition* was revealed by the previous ANOVA, we averaged PF‐W and PF‐PW conditions in a letter strings condition (PF‐LE).

In grade 1, there was a main effect of *Hemisphere* (*F*_1,38_ = 4.63, *p* = .038, η^2^ = 0.11), showing larger responses in the LH (2.00 µV) than in the RH (1.62 µV), a significant interaction between *Hemisphere* and *Electrode Position* (*F*_1,38_ = 7.61, *p* = .009, η^2^ = 0.17), and no main effect of *Electrode Position* (*F* < 1). Inter‐hemispheric paired comparisons for each electrode position showed that responses in the LH were significantly larger than in the RH for posterior electrodes (O1 vs. O2: (*t*[38] = 3.34; *p* = .002), while LH and RH responses did not differ on lateral electrodes (P7 vs. P8: (*t*[38] = 0.35; *p* = .730)). In grade 2, there was a main effect of *Hemisphere* (*F*_1,38_ = 11.88, *p* = .001, η^2^ = 0.24), responses were larger in the LH (3.78 µV) than in the RH (2.82 µV). There were no other main effects or interactions (all *F*s < 1) (Figure 3).

##### Gain scores and topographical change

**FIGURE 4:**
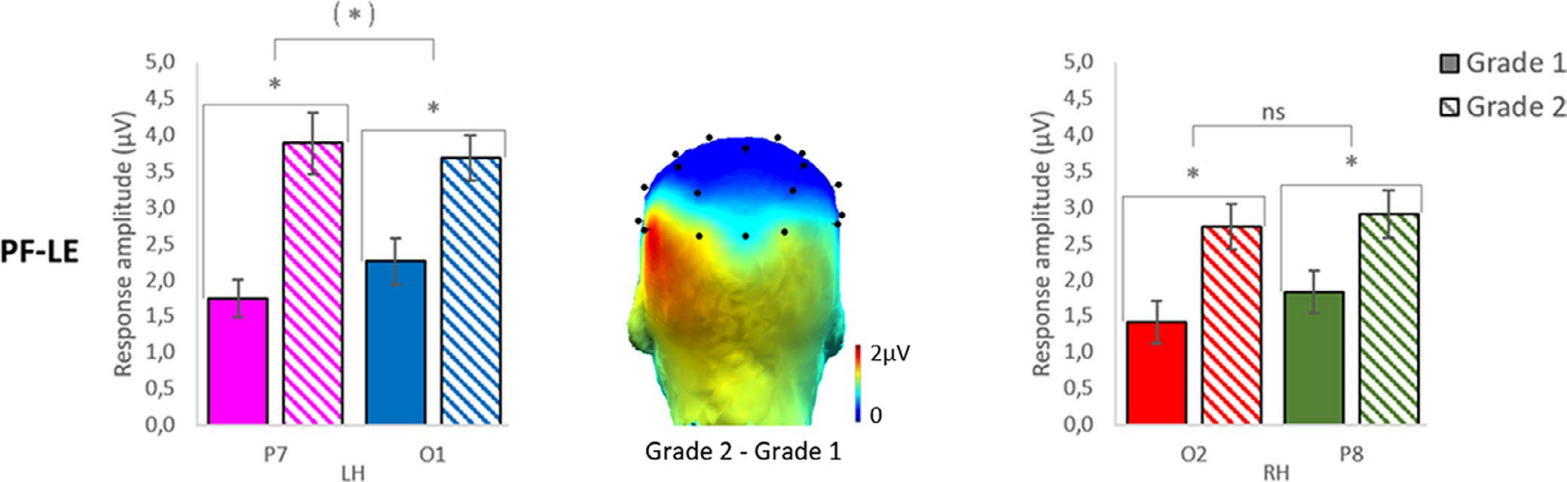
Response pattern evolution between grade 1 and grade 2 for letter strings. Bar graphs, with standard errors of the mean, display the sum of baseline subtracted amplitudes computed on five significant harmonics in each grade (grade 1: plain bars; grade 2: dashed bars) and hemisphere (left‐LH, right‐RH) for letter strings (mean words and pseudowords) within pseudofont strings. The response amplitude significantly increases from grade 1 to grade 2 for each electrode but amplitude gains were higher in the LH than the RH. In the LH, the gain tended to be greater on the left lateral electrode P7 (*p* = .05) than on the left posterior electrode O1. In the RH, amplitude gains did not differ according to electrode position. The scalp topography represents the amplitude gain between grade 1 and grade 2, which is greatest on the left lateral electrode P7

To better qualify the evolution from grade 1 to grade 2, we computed gain scores by subtracting the response amplitudes of grade 1 from the response amplitudes of grade 2 (grade 2—grade 1; see topography of amplitude gain in Figure 4). All gain scores were significantly different from 0 (all *p* values = .000).

An ANOVA was performed on gain scores with *Electrode Position* (posterior‐O1/O2, lateral‐P7/P8) and *Hemisphere* (LH, RH) as within‐subjects factors. It showed a main effect of *Hemisphere* (*F*_1,38_ = 6.31, *p* = .016, η^2^ = 0.14), with higher gains in the LH (1.78 µV) than in the RH (1.20 µV) and a significant interaction between *Electrode Position* and *Hemisphere* (*F*_1,38_ = 4.28, *p* = .045, η^2^ = 0.10). In the LH, gains tended to be greater (*t*[38] = 1.98; *p* = .055) on the left lateral electrode P7 (2.14 µV) than on O1 (1.42 µV). In the RH, increases did not differ according to electrode position (*t*[38] = 0.99; *p* = .327), P8 (1.07 µV) and O2 (1.32 µV) (Figure 4).

##### Brain‐behavior correlations

**FIGURE 5:**
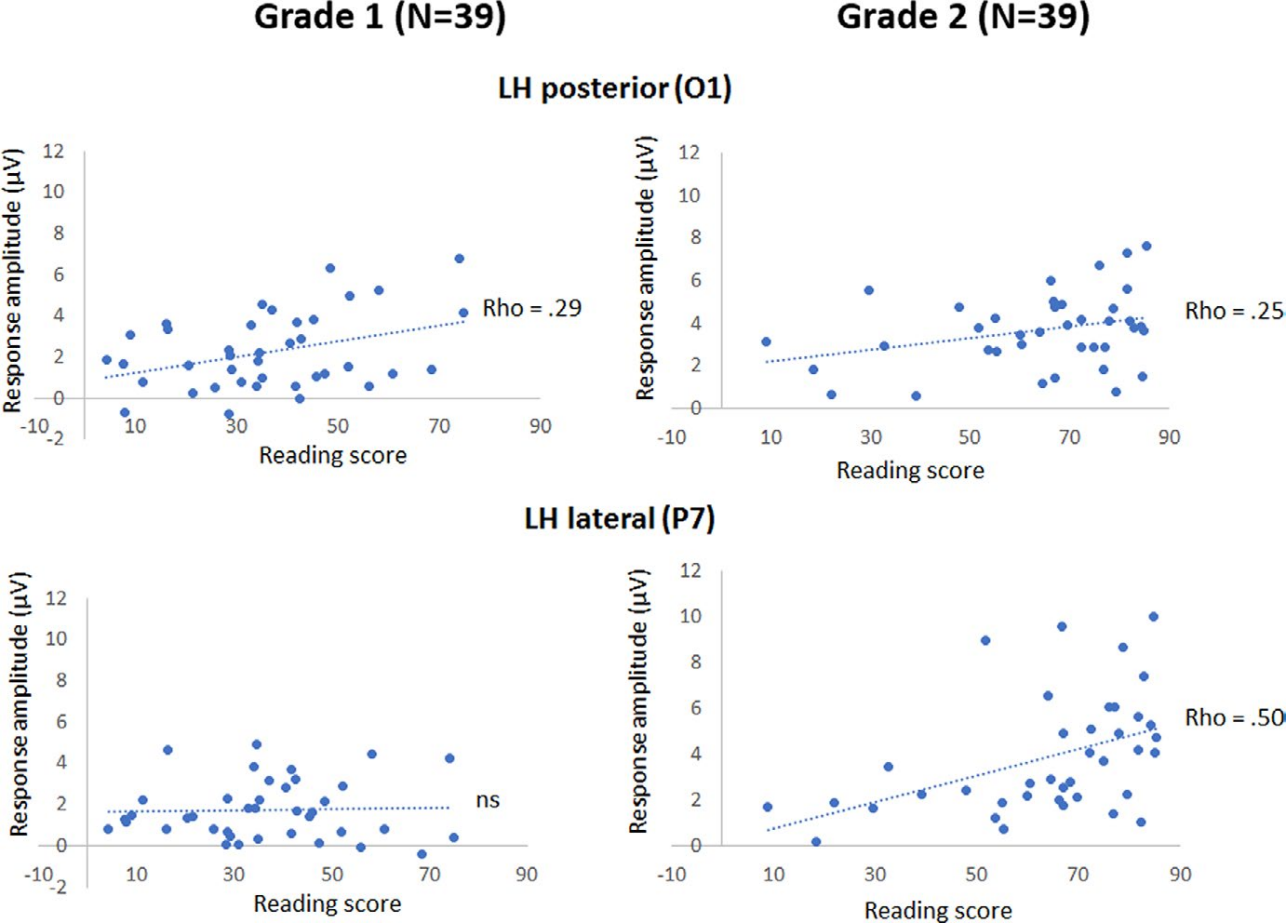
Significant correlations between reading composite scores (accuracy in %) and the response amplitudes for letter strings within pseudofont strings (PF‐LE condition) in each grade. In grade 1, a positive correlation was observed with response amplitudes on O1 only, while in grade 2, a positive correlation was observed with response amplitudes on P7

Non‐parametric correlations (Kolmogorov‐Smirnov test for reading composite scores: *p* > .050 in grade 1 but *p* < .050 in grade 2) were ran between the composite reading score and the four electrodes selected above (P7 and O1 in the LH; P8 and O2 in the RH). These analyses revealed a relationship between reading scores and responses to letter strings. In grade 1, composite reading scores correlated with response amplitudes on O1 only (Spearman Rho = 0.29; *p* = .035) while in grade 2, composite reading scores correlated with response amplitudes on P7 (Rho = 0.50; *p* = .001) and tended to correlate with response amplitudes on O1 (Rho = 0.25; *p* = .059). No correlation was found with response amplitudes in the RH (Figure 5). However, after Bonferroni correction for multiple comparisons, only the correlation of reading scores with P7 in grade 2 remained significant (at an alpha level of *p* = .0124). Replicating these analyses without the regular/irregular word reading subtests (see Methods) yielded identical results (see Supporting Information, Analysis 2).

##### Response distribution over harmonics

**FIGURE 6:**
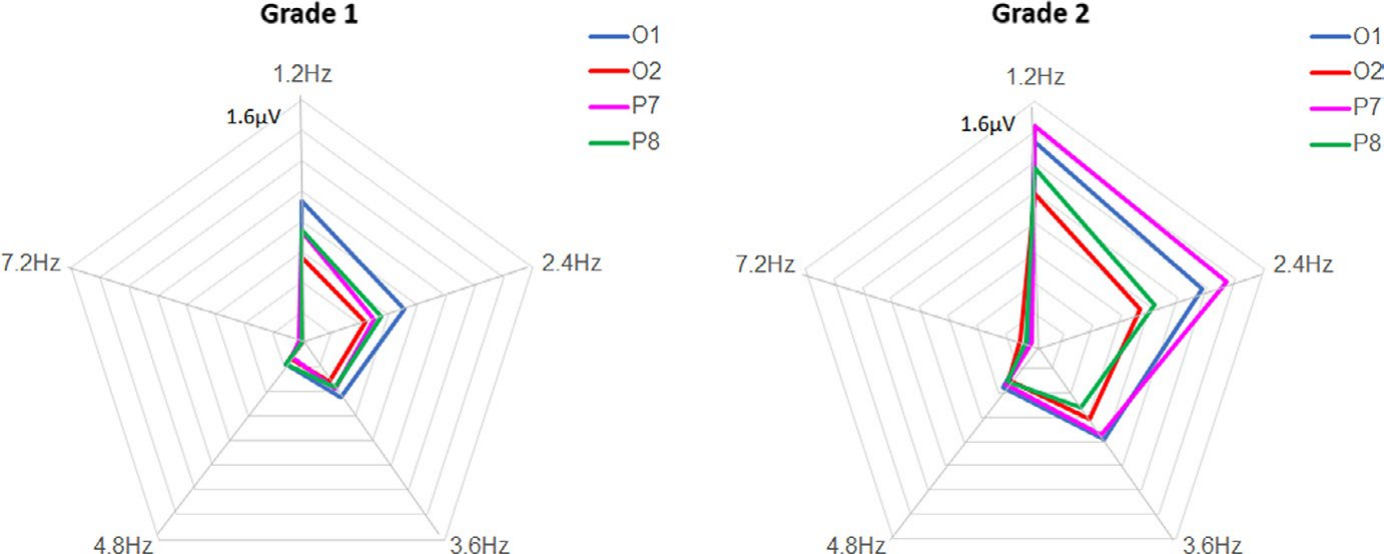
Fingerprint diagram of the harmonic frequency amplitude distribution in each grade for discrimination responses of letter strings within pseudofont strings (PF‐LE condition). Baseline subtracted amplitudes of the five significant harmonics (from F/5 or 1.2 Hz to 6F/5 or 7.2 Hz, excluding the base stimulation frequency; see Methods) are plotted for each grade and electrode of interest as vectors of the diagrams. The left electrode P7 showed the clearest changes: there was a relative decrease of 5% on the first harmonic, but an increase of up to 6% on the second harmonic

We observed that the responses tended to be relatively more distributed across harmonics in grade 2 than in grade 1. To explore this possibility, we plotted for each grade and each electrode of interest the amplitude values as vectors of a fingerprint diagram (Figure 6). This figure suggests that the second harmonic became slightly more important in grade 2 than in grade 1 for left electrodes O1 and P7. When computed in percentage of the total response, there was a very slight decrease of 5% on the first harmonic, and an increase of up to 6% on the second harmonic on P7 (see Table S1—Supporting Information).

#### 3.2.3 Response pattern and evolution for symbol strings

##### Response amplitudes

**FIGURE 7:**
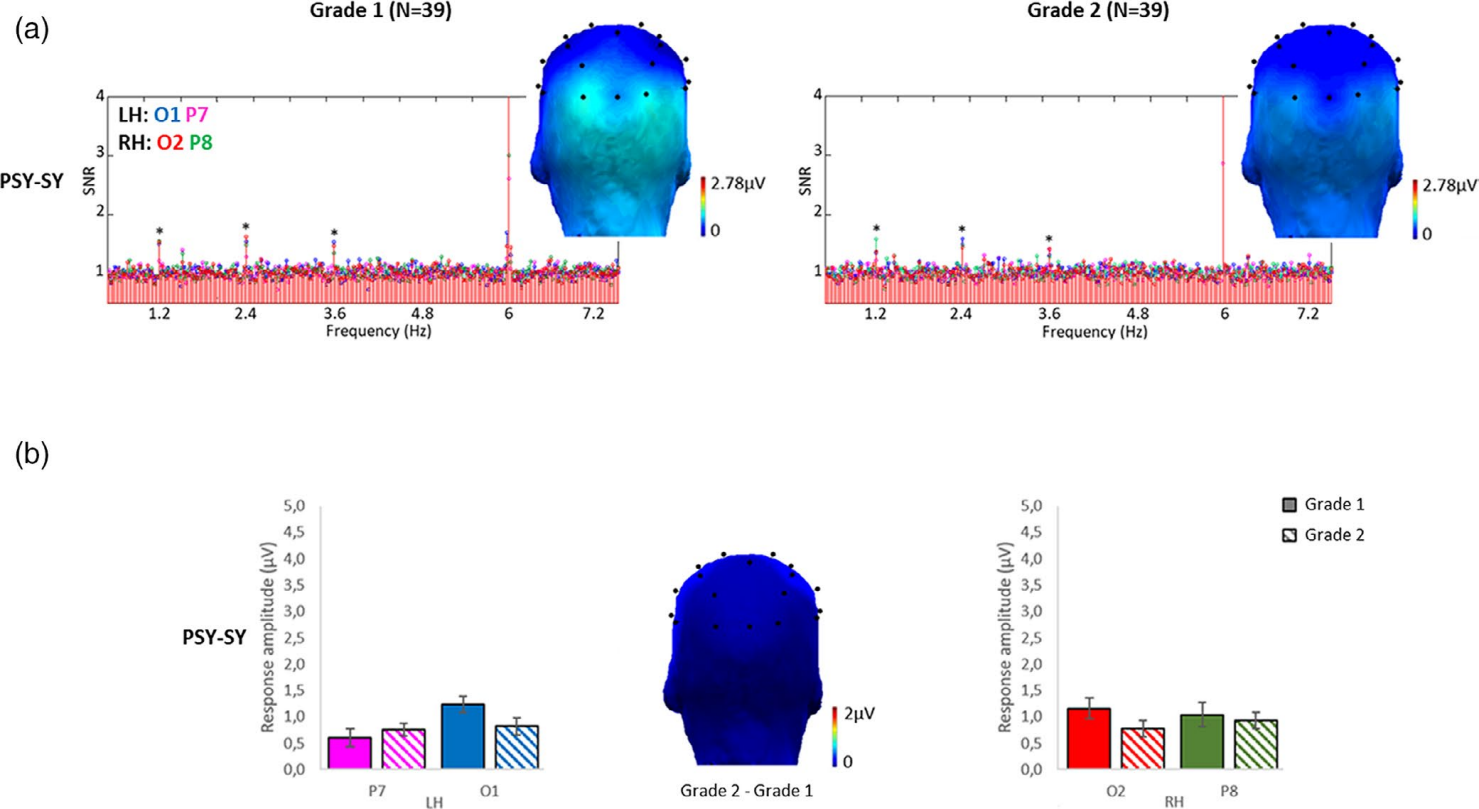
Discrimination responses to symbol strings in each grade. (a) Grand‐averaged (*N* = 39) SNR EEG spectra on O1 (blue), P7 (magenta), O2 (red) and P8 (green) and scalp topographies (sum of baseline subtracted amplitudes computed on five significant (*) harmonics except the base stimulation frequency; see Methods). In each grade (columns), there were significant discrimination responses (*Z* scores > 2.58) to symbol strings within pseudosymbol strings (PSY‐SY condition) from F/5 or 1.2 Hz to 3F/5 or 3.6 Hz. (b) Bar graphs, with standard errors of the mean, display response amplitudes of each grade (grade 1: plain bars, grade 2: dashed bars) (sum of baseline‐subtracted amplitudes computed on five significant harmonics). On the contrary to letter strings condition, the scalp topography (the amplitude of grade 2 minus the amplitude of grade 1) showed no amplitude gain between grades (there was only a slight decrease of response amplitude on posterior electrodes)

For the control condition PSY‐SY, an ANOVA was performed on discrimination responses with *Grade* (grade 1, grade 2), *Hemisphere* (LH, RH) and *Electrode Position* (posterior‐O1/O2, lateral‐P7/P8) as within‐subjects factors. It showed only a significant interaction between *Grade* and *Electrode Position* (*F*_1,38_ = 9.24, *p* = .004, η^2^ = 0.20) (no main effect or any other interaction; all *F*s < 1). In grade 1, the posterior response (mean O1/O2: 1.20µV) was larger (*t*[38] = 2.60; *p* = .013) than the lateral response (mean P7/P8: 0.82 µV) while in grade 2, response amplitudes did not differ (*t*[38] = 0.40; *p* = .69) between posterior (0.80 µV) and lateral (0.84 µV) responses. This evolution was due to a slight decrease of response amplitudes on posterior electrodes between first and second grade (gain scores computation differed from 0 for the posterior response only: (*t*[38] = 2.09; *p* = .043) (Figure 7).

##### Brain‐behavior correlations

Composite reading scores did not significantly correlate with response amplitudes to symbol strings in grade 1 (O1: Spearman Rho= −0.26; *p* = .058, P7: Rho = 0.03; *p* = .427, O2: Rho=−0.24; *p* = .074, P8: Rho = 0.01; *p* = .470), nor in grade 2 (O1: Rho = 0.09; *p* = .295, P7: Rho=−0.05; *p* = .376, O2: Rho = 0.08; *p* = .310, P8: Rho=−0.07; *p* = .325). Replicating these analyses without the regular/irregular word reading subtests yielded identical results (see Supporting Information, Analysis 3).

### 3.3 Sensitivity at the individual level

Since one of the strengths of the FPVS‐EEG approach is its sensitivity at the individual level, we determined how many children showed a clear left‐lateralized response to letter strings. In addition, we determined how many showed a statistically significant letter strings‐selective response in each grade.

**FIGURE 8:**
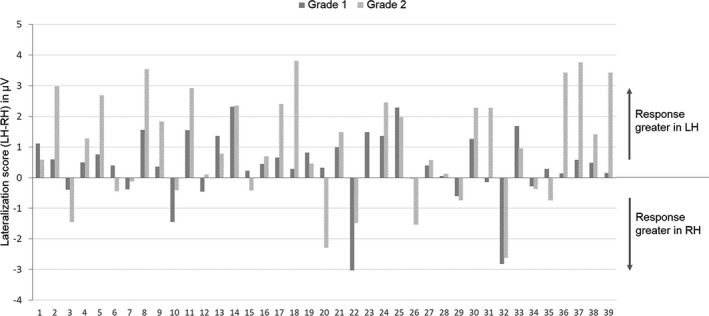
Individual lateralization scores in each grade for responses to letter strings within pseudofont strings (PF‐LE condition). The histogram plots the difference in response amplitudes in the left hemisphere (LH) minus response amplitudes in the right hemisphere (RH) (LH‐RH in microvolts). 62% of the children are left‐lateralized at both grades

First, we computed individual lateralization scores by subtracting the response amplitude in the RH (mean O2, P8) from the response amplitude in the LH (mean O1, P7) (LH‐RH; positive values reflect left‐lateralization, negative values reflect right‐lateralization and values equal to zero reflect bilateral responses). At the individual level, the LH dominance was present in the majority of children: 24 out of 39 were left‐lateralized (62%) while only 8 were right‐lateralized in both grades (21%, one of whom was left‐handed), and the others showed a change in lateralization pattern across grades (Figure 8). For children who were left‐lateralized in both grades, the amplitude of the left‐lateralization increased significantly on average from grade 1 (0.91 µV, *SD*: 0.65) to grade 2 (1.98 µV, *SD*: 1.18) (*t* = −3.789; *p* = .001): although responses increased in both hemispheres, it did so proportionally more in the LH (LH: from 2.37 to 4.35 µV; RH: from 1.48 to 2.35 µV). For children who were right‐lateralized, the amplitude of the right‐lateralization remained stable (grade 1:1.12 µV, *SD*:1.18; grade 2:1.09 µV, *SD*: 0.83, *t* = −0.074; *p* = .94), responses increasing similarly in both hemispheres (LH: from 1.32 to 3.35 µV; RH: from 2.45 to 4.45 µV).

**FIGURE 9:**
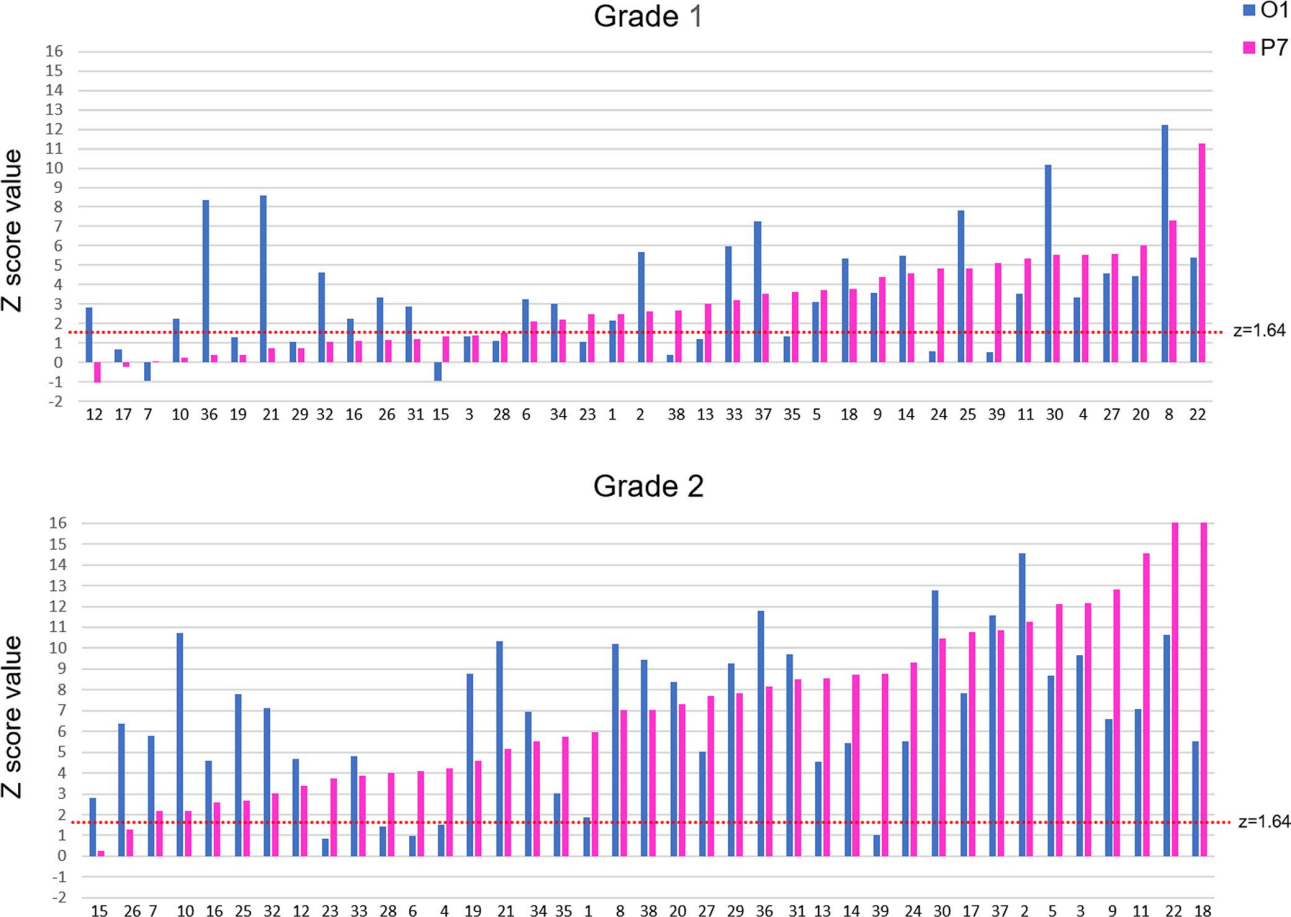
Individual *Z* score values in each grade for responses to letter strings within pseudofont strings (PF‐LE condition). Children are displayed as a function of ascending *Z* score values on the electrode P7 (magenta), and their score on electrode O1 is also displayed (blue). At a threshold of Z = 1.64 (*p *< .05; represented by the red dotted line), 82% of first graders and 100% of second graders have a significant response for letters on P7 and/or O1

Second, we computed individual *Z* scores (see Methods) that are reported in Figure 9. In both grades, children were plotted on the *X*‐axis as a function of ascending values in *Z* score for the electrode P7. Considering a *Z* score value of 1.64 as threshold (*p *< .05; Figure 9, dotted red line), 18/39 (46%) of children in grade 1 have a significant response on both O1 and P7, 8/39 (21%) have a significant response on O1 and 6/39 (15%) have a significant response on P7. Among the 7/39 remaining children, 5 have reading scores below the group average (36.45% in composite reading score). In grade 2, results show a significant LH response in every child, with 37/39 (95%) of children displaying a significant response on P7, and 2/39 (5%) on O1. Even at a conservative threshold of Z = 3.1 (*p* < .001, one‐tailed), in grade 2 all children except one (98%) show a significant letter‐selective response on at least one of these two LH electrodes. The single child without significant responses at this threshold had one of the lowest reading scores of the group (18% in composite reading score vs. 63.8% for the group average).

Third, we calculated the number of children who showed a shift in topographical response between O1 and P7 on the sum of baseline subtracted amplitudes, as found at the group‐level. In grade 1, 64% (25/39) of children showed a larger response on O1 than on P7 while only 31% (12/39) of children showed a larger response on P7 than on O1. In grade 2, almost the same proportion of children showed a larger response on O1 (44%; 17/39) or on P7 (51%; 20/39). Considering amplitude increases from grade 1 to grade 2, 34/39 (87%) of children showed larger responses on P7, and 26/39 (67%) showed amplitude increases on O1.

## 4 DISCUSSION

The current longitudinal study assessed the evolution of neural tuning to letter strings and its relationship with emerging reading abilities. Thirty‐nine children were tested twice, at the beginning (i.e. during the first trimester of grade 1) and after 1 year of formal reading instruction behaviorally and with FPVS‐EEG. FPVS‐EEG is an approach that rapidly (here, 3 × 40 s of recording per condition) measures automatic and selective visual discrimination responses with high sensitivity at the individual level (Guillaume et al., 2018; Liu‐Shuang et al., 2014; Lochy et al., 2015) and high test‐retest reliability (Dzhelyova et al., 2019). Our findings evidenced a clear left lateralized letter selectivity in both grade 1 and grade 2 independently of lexical access. Following 1 year of schooling, letter selectivity evolves, both quantitatively and qualitatively within the LH. Remarkably, in both grades, neural responses were highly significant at the individual level. These findings will be discussed in turn.

Already at the first trimester of grade 1, our results show clear left lateralized letter selectivity, despite the limited reading ability (36.45% accuracy in composite reading score; single letter knowledge: 19/26 ± 5 *SD*; see Table 1). This agrees with recent FPVS‐EEG (Lochy et al., 2016) or fMRI (Dehaene‐Lambertz et al., 2018) studies and confirms that letter selectivity in the LH emerges earlier (Lochy et al., 2016) than initially proposed in other EEG (N1) studies on reading acquisition (Maurer et al., 2005, 2006), where it was hypothesized to emerge after 2–2.5 years of reading instruction. The present finding also contrasts with EEG studies that report neural tuning to letters to be bilateral at the end of first grade (Eberhard‐Moscicka et al., 2015) or even right‐lateralized in preschool children (Maurer et al., 2005). On the contrary, it supports the proposal that learning to associate letters with speech sounds starts very early on to trigger connections between posterior visual regions and anterior language regions (Brem et al., 2010; Karipidis et al., 2018; Maurer & McCandliss, 2007; Pleisch et al., 2019), or alternatively, that there exist pre‐wired connections between these regions (Saygin et al., 2016; Stevens et al., 2017).

Therefore, our findings contradict the view that letters are first processed as familiar visual shapes, involving mainly RH object recognition brain structures, before becoming linked to phonology and language (Maurer et al., 2006), although this does not exclude the possibility that at later stages of reading (words), language transparency might modulate the involvement of the RH. This view concerns at least the age range tested until now with FPVS‐EEG (5 years old in Lochy et al., 2016; 6 and 7 years old here). Whether, at a younger age, letters could be processed as familiar shapes by the RH, without any phonological context (e.g. with mere exposure in preschool classrooms), remains to be determined. Then, at a later developmental stage of word reading, one might again expect a differential engagement of the LH (for decoding predictable GP patterns, i.e. regular words) and of the RH (for strings where mapping phonology to orthography is indirect and involve some rote‐visual learning; i.e. irregular words). Lexical access was neither found in grade 1 nor in grade 2. This suggests that selective neural responses to words requires a high level of word recognition automatization that is not related to letter selectivity or to early reading abilities at the behavioral level (Coch & Meade, 2016; Eberhard‐Moscicka et al., 2015; Zhao et al., 2014).

After 1 year of reading instruction, response amplitudes of letter selectivity showed a remarkable quantitative increase, with 93% stronger responses in the LH (63% on O1 and 122% on P7). These increases appear to be specific to letters: they are not related to a general increase of visual stimulation responses and not observed with discrimination responses to familiar symbol strings. While discrimination responses increased only for letter selectivity (PF‐W and PF‐PW), base rate responses increased in all conditions with a similar proportion across all four conditions (46% for PF‐LE, and 42% for PSY‐SY). Even for the PW‐W condition, where no lexical discrimination response was observed, the base rate response was significant and increased after 1 year of schooling. Furthermore, for PF‐LE condition, base rate responses increased relatively less (44% increase) than discrimination responses (93% increase). This supports the notion that base rate responses and responses to deviant stimuli reflect different types of processes (neural responses to the visual periodic stimulation vs. neural discrimination of deviant stimuli).

Concerning the discrimination responses of familiar symbol strings, at both testing sessions, they were weak and bilateral, and they decreased between grade 1 and grade 2 on both left (O1) and right (O2) posterior sites. This implies that the increase in response amplitude observed for letter strings does not merely reflect general age‐related changes (e.g. at the anatomical level, at the functional level, or from better attentional abilities) or visual familiarity. In line with this view, while composite reading scores correlated with left response amplitudes for letter strings (reliably in grade 2), no correlation was found between reading composite scores and response amplitudes for symbol strings. Curiously, this bilateral response differs from a previous study in 5‐year‐old preschool children who instead presented with a right lateralized response to symbol strings (Lochy et al., 2016), interpreted as reflecting visual familiarity. We can only speculate at this stage that between preschool and the beginning of grade 1, children might have associated to the symbols some knowledge beyond pure visual familiarity, like verbal labels, therefore displaying a slight shift of the responses towards the LH. Importantly, as the orthogonal fixation task revealed an accuracy almost at ceiling for both testing sessions, the differences observed between neural discrimination responses of letter and symbol strings are not due to a fluctuation of attention between conditions.

After 1 year of reading instruction, qualitative changes in letter selectivity were also observed. They appeared mainly as a topographical change: responses to letter strings evolved from posterior middle occipital electrode (O1) to a more lateral electrode (P7) within the LH. In line with this finding, brain‐behavior correlations between composite reading scores and discrimination responses to letters evolved from a moderate, though non‐significant correlation with O1 (Rho = 0.29) in grade 1 to a stronger and more reliable correlation with P7 in grade 2 (Rho = 0.50). This topographical change could reflect either a change in the inner sources of the response, or a change due to anatomical growth, or furthermore an increase in signal that spreads to other electrodes on the scalp.

Other observations in neuroimaging described responses to letter strings (VWFA) to be more posterior in children (10.2 ± 3.0 years old) than in adults (Olulade et al., 2013). Preschool children (1 year younger than the sample of the current study at time 1, Lochy et al., 2016) also displayed letter‐selective responses on the left posterior electrode O1 when tested with the same approach as here, while in adults, the maximal response was on occipito‐temporal electrode PO7 (Lochy et al., 2015). However, in adults, the stimuli used were words among pseudofont strings, and word‐recognition is automated in adults. Previous studies have described slightly different regions in the VOTC for processing letters (more posterior) versus well‐formed letter strings (words or pseudowords, more anterior), either with fMRI (in 10.1 ± 2.9–11.3 ± 0.4 years old children: Brem et al., 2009; Olulade, Flowers, Napoliello, & Eden, 2015; Van der Mark et al., 2009 and in adults: Thesen et al., 2012; Vinckier et al., 2007) or with intracerebral recordings (Lochy et al., 2018; Thesen et al., 2012). However, the young children tested here do not yet automatically recognize words, therefore it does not seem very plausible that anterior brain structures related to word‐recognition would be the origin of the responses in grade 2 and not in grade 1. A second possibility would be that the change in topography reflects general effects due to growth‐related anatomical changes, for instance in skull thickness or gyri/sulci differences in growth/orientation. However, we should then also have observed a change in scalp topography for the response to familiar symbol strings, which is not the case.

Finally, the topographical change measured on the scalp could also reflect an increase in the same posterior neural population response, with a change of orientation of the intracerebral sources. This would be compatible with the current view in the literature, where fMRI longitudinal studies show an increase in amplitude of signal responses in VOTC with age and behavioral improvement in reading ability (Ben‐Shachar et al., 2011; Booth et al., 2001; Dehaene‐Lambertz et al., 2018; Turkeltaub et al., 2008). It is also in line with studies showing that the magnitude of BOLD signal change for letter‐*specific* responses is greater in adults than in children (7–14 years old) (Centanni et al., 2017). Interestingly, when comparing letter‐s*pecific* responses (letters vs. faces) to letter‐s*elective* responses (letters vs. false fonts or line drawings), only the latter varied in intensity between children and adults and correlated with reading ability (Centanni et al., 2017, 2018), corresponding to the same level of letter‐selectivity that we tested here when measuring discrimination of letters among pseudo‐fonts.

The second aspect of qualitative change that we examined is derived from previous studies comparing infants to young children and adults (Lochy, de Heering, et al., 2019), or children in different tasks implying various discrimination levels (Lochy, Schiltz, & Rossion, 2019), which suggested that the distribution of responses across harmonics is informative of the complexity of the response in the time‐domain. Indeed, the simplest response, a perfect sinusoid, would concentrate its power in the frequency‐domain in only one harmonic, while more complex responses, with sharper edges (rise/decay differing from a sinusoid) distribute over multiple harmonics (Regan, 1989; Zhou, Melloni, Poeppel, & Ding, 2016). Here, we expected that discrimination responses would be more distributed in grade 2 than in grade 1, but the trend was only weak on overall electrodes (−3% on the first harmonic and +0% on the second one). Although this change was somewhat clearer on electrode P7 (−5% on the first harmonic and +6% on the second harmonic), this aspect of qualitative change in response patterns was not well supported in the present study with children 1 year apart, and has to be investigated in future studies with older children.

Finally, the FPVS neural tuning index was highly sensitive at the individual level. When examining left‐lateralization beyond the group level, it was observed in 62% of children (21% were right‐lateralized, while the others showed no hemispheric preference). The amplitude increase in the LH for those left‐lateralized children was clearly greater (1 µV) than for right‐lateralized children where it did not change. When examining individual *Z* scores, 82% of children in grade 1 had a significant response on one of the two left electrodes (O1, P7), and in grade 2, virtually all of them displayed a significant response. When examining the shift in topographical response between O1 and P7, 64% of children showed a larger response on O1 and only 31% of children showed a larger response on P7 in grade 1 while in grade 2, almost the same proportion of children showed a larger response on O1 (44%) or on P7 (51%).

The advantages of the FPVS‐EEG approach for studying children longitudinally are substantial in terms of objectivity (i.e. behavior‐free; responses expected at experimentally defined frequencies), selectivity (i.e. specificity) and Signal to Noise Ratio (i.e. sensitivity) (Rossion, 2014). The increased sensitivity relates to the fast and uninterrupted stimulation, where each stimulus is forward and backward masked by previous/next stimuli. This approach is a measure of automatic visual discrimination processes, as it reflects an index of differential processing between two categories (no need to perform a comparison or ‘cognitive subtraction’ between two processes or categories, increasing its sensitivity). The associated reduction of measurement time is a considerably asset for developmental studies.

Altogether, the current longitudinal study, conducted in natural settings, provides important new elements for understanding the developmental course of early neural tuning for letter strings in typically developing children. This study also highlights the potential of FPVS‐EEG measures to be applied in understanding early neurobiological processes of reading acquisition, at the stage of letter selectivity, thus before the automatization of GP mappings ability and word recognition. Developing such measures is crucial both for educational and clinical outcomes.

## CONFLICT OF INTEREST

None.

## Supporting information

Supplementary Material

Video S1

## Data Availability

The data that support the findings of this study are available from the corresponding author upon request.
