# Supplementary material for: Developmental changes in neural letter‐selectivity: A 1‐year follow‐up of beginning readers
Source: Dev Sci. 2020 Jun 10;24(1):e12999. doi: 10.1111/desc.12999 (PMC7816260; doi:10.1111/desc.12999)
Supplement: Supplementary file 1 — Supplementary Material [file DESC-24-e12999-s001.docx]

**Supporting Information**

**Supplementary Video 1.** 22 s excerpt of a stimulation sequence, showing pseudofont strings at 6Hz, with (french) words (PF-W condition) appearing every five items (i.e., 1.2Hz). The video 1 was generated with a JavaScript (Java SE Version 8) in the MP4 format.

==============================INSERT VIDEO HERE=====================================

**Supplementary Figure 1.** Electrodes of interest selection for base rate responses (referring to point 1.1.).

| 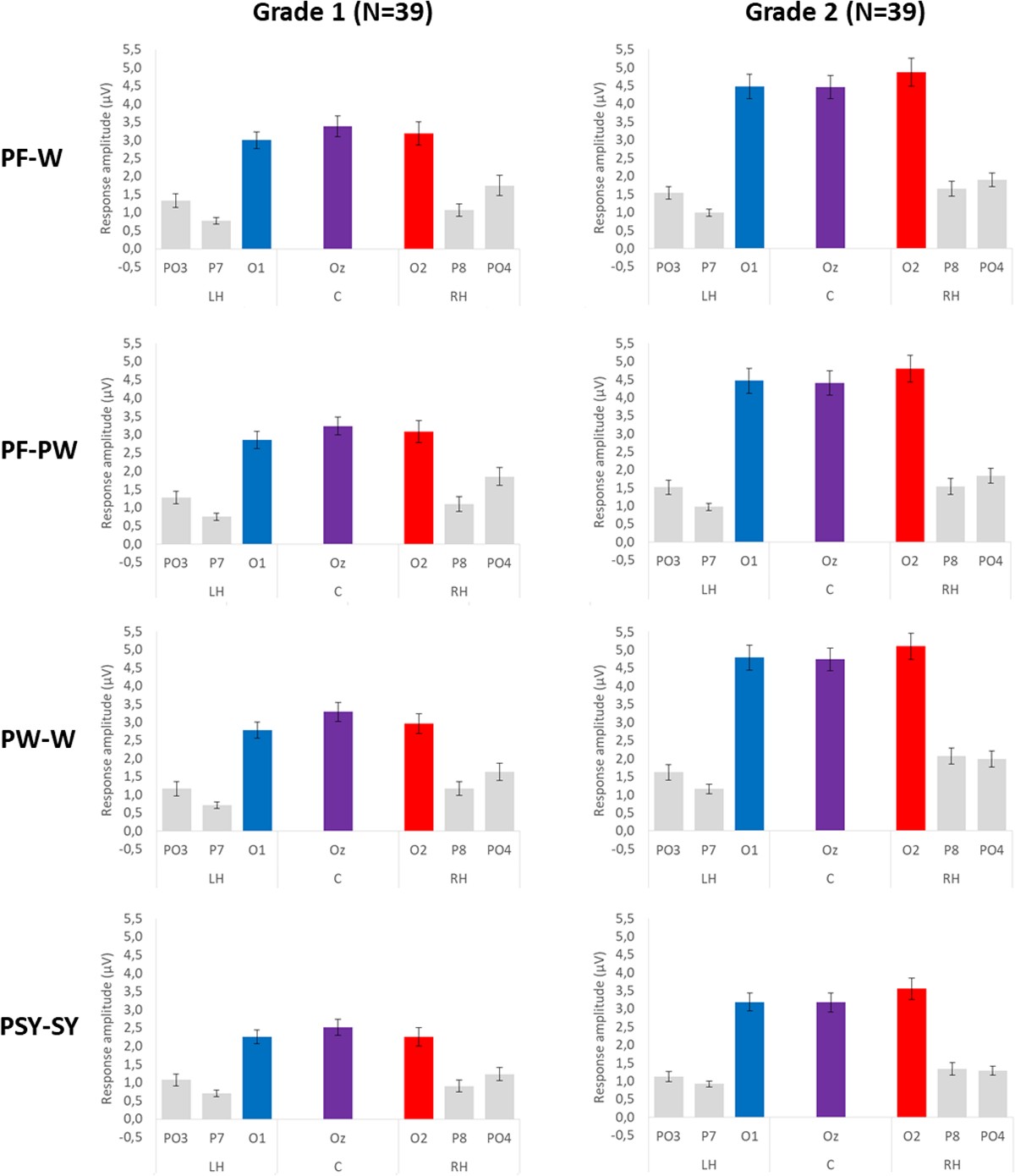 |
| --- |
| **Figure S1. Electrodes of interest selection for base rate responses.** In each condition (in rows) and grade (in columns), bar graphs, with standard errors of the mean, represent the sum of baseline subtracted amplitudes for the highest number of significant harmonics found across conditions and grades (from 6Hz to 42Hz, see Methods). In all conditions and in both grades, the largest response was recorded at three middle occipital (MO) electrodes, O1, Oz  and O2. |

**Supplementary Figure 2.** Electrodes of interest selection for discrimination responses of deviant stimuli (referring to point 2.1.).

| 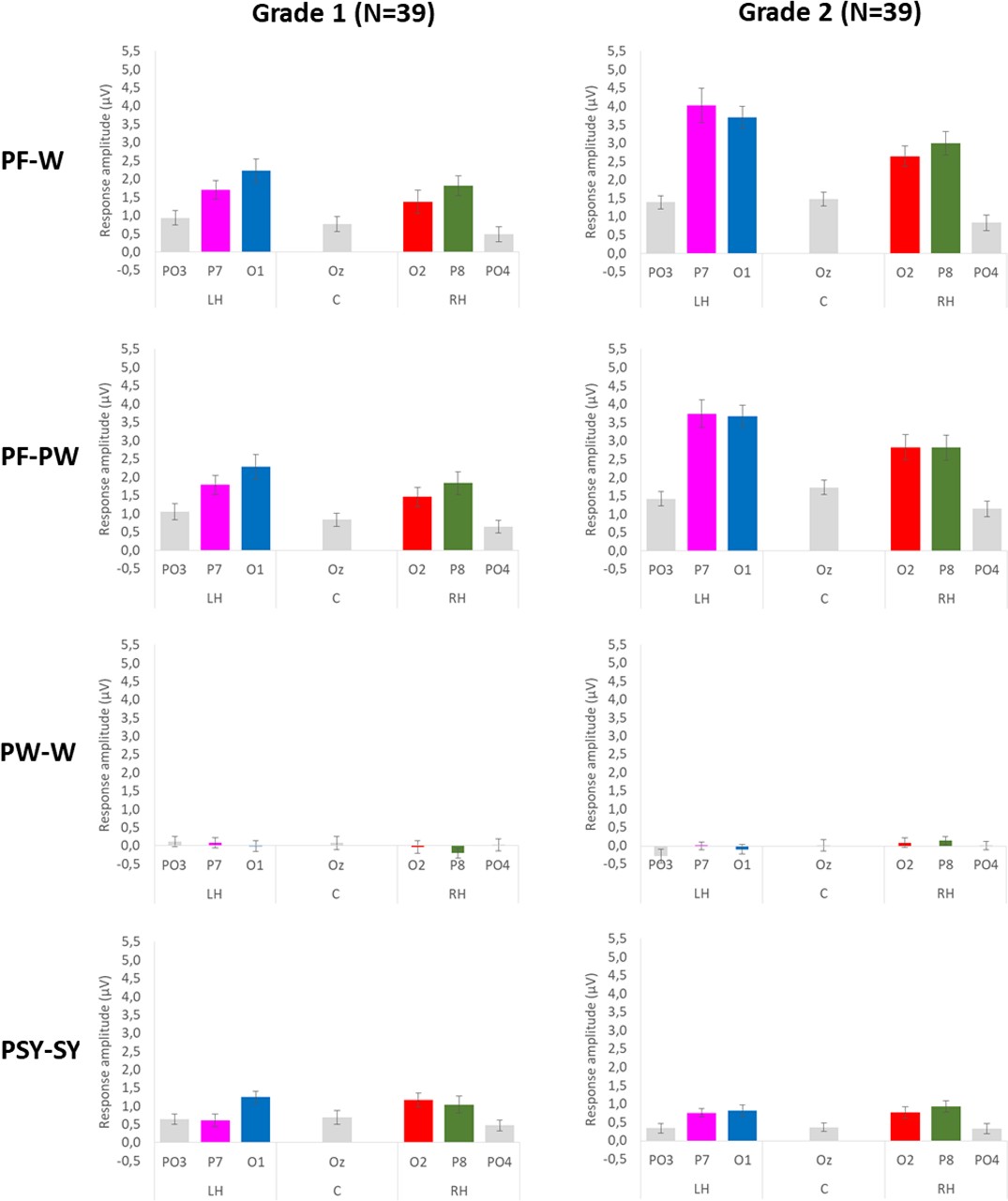 |
| --- |
| **Figure S2. Electrodes of interest selection for discrimination responses of deviant stimuli.** In each condition (in rows) and grade (in columns), bar graphs, with standard errors of the mean, represent the sum of baseline subtracted amplitudes for the highest number of significant harmonics found across conditions and grades (from 1.2Hz to 7.2Hz excluding 6Hz, see Methods). Except for PW-W condition that showed no significant discrimination response, the largest responses were recorded on the left O1 and P7 postero-lateral  electrodes as well as on their homologous right postero-lateral electrodes O2 and P8. |

**Supplementary Analysis 1.** Discrimination responses for all letter strings conditions; PF-W, PF-PW, PW-W (referring to point 2.2.)

An ANOVA was performed on the sum of baseline subtracted amplitudes of responses to letter strings with *Grade* (G1, G2), *Condition* (PF-W, PF-PW, PW-W), *Hemisphere* (LH, RH) and *Electrode Position* (posterior-O1/O2, lateral-P7/P8) as within-subjects factors. There was a significant main effect of *Hemisphere* [F1,38 = 10.21, *P* = 0.003, η² = .21], responses being higher in the LH (1.93µV) than in the RH (1.48µV), a main effect of *Grade* [F1,38 = 60.74, *P* = 0.000, η² =

.61], responses being overall higher in grade 2 (2.21µV) than in grade 1 (1.19µV) and a main effect of *Condition* [F2,76 = 128.76, *P* = 0.000, η² = .77], responses to PW-W condition being lower (-0.002µV) than PF-W (2.56µV) and PF-PW (2.55µV), which did not differ from each other. There was also a significant interaction between *Grade* and *Condition* [F2,76 = 13.99, *P* = 0.000, η² = .27], between *Condition* and *Hemisphere* [F2,76 = 8.35, *P* = 0.002, η² = .18] and between *Grade*, *Condition* and *Hemisphere* [F2,76 = 6.26, *P* = 0.005, η² = .14] qualified by a significant quadruple interaction between *Grade*, *Condition*, *Hemisphere* and *Electrode Position* [F2,76= 3.35, *P* = 0.040, η² = .08]. There were no other main effects or interactions (all Fs <1).

For each grade, ANOVAs were performed on the sum of baseline subtracted amplitudes with *Condition* (PF-LE-mean PF-W/PF-PW, PW-W), *Hemisphere* (LH, RH) and *Electrode Position* (posterior-O1/O2, lateral-P7/P8) as within-subjects factors. In grade 1, there was a significant main effect of *Condition* [F1,38= 60.69, *P* = 0.000, η² = .62], responses being higher in PF-LE condition (1.81µV) than PW-W condition (-0.04µV) and a main effect of *Hemisphere* [F1,38= 6.48, *P* = 0.015, η² = .15], responses being higher in the LH (1.02µV) than in the RH (0.75µV). There was also a significant triple interaction between *Condition*, *Hemisphere* and *Electrode Position* [F1,38= 7.78, *P* = 0.008, η² = .17]. In order to decompose the triple interaction between *Condition*, *Hemisphere* and *Electrode Position*, ANOVAs were performed by condition with *Hemisphere* (LH, RH) and *Electrode Position* (posterior-O1/O2, lateral-P7/P8) as within- subjects factors. For PF-LE condition, there was a significant main effect of *Hemisphere* [F1,38= 4.63, *P* = 0.038, η² = .11], responses were higher in the LH (2µV) than in the RH (1.62µV) and a significant interaction between *Hemisphere* and *Electrode Position* [F1,38= 7.61, *P* = 0.009, η² =

.17]. Inter-hemispheric paired comparisons for each electrode position showed that responses in the LH were significantly higher than in the RH for posterior electrodes (O1 vs O2: [*t*(38) =

3.34 ; *P* = 0.002], while LH and RH responses did not differ on lateral electrodes (P7 vs P8: [*t*(38) = 0.35 ; *P* = 0.730]). For PW-W condition, there were no main effects or interactions (all Fs <1). In grade 2, there was a significant main effect of *Condition* [F1,38= 188.64, *P* = 0.000, η²

= .83], responses were higher in PF-LE condition (3.30µV) than in PW-W condition (0.04µV) and a main effect of *Hemisphere* [F1,38= 7.34, *P* = 0.010, η² = .16], responses were higher in the LH (1.87µV) than in the RH (1.47µV). There was also a significant interaction between *Condition* and *Hemisphere* [F1,38= 13.98, *P* = 0.001, η² = .27]. For PF-LE condition, inter- hemispheric paired comparisons showed that responses in the LH (3.78µV) were higher [*t*(38) =

3.45 ; *P* = 0.001] than in the RH (2.82µV). For PW-W condition, there was no difference [*t*(38) =

1.53 ; *P* = 0.136] between the LH and the RH.

**Supplementary Analysis 2.** Replication of brain-behavior correlations between composite reading scores and discrimination responses of letter strings (referring to point 2.2.3.), excluding regular/irregular word reading (subtest for which items differed between grade 1 and grade 2)

The relationship between composite reading scores and responses to letter strings (baseline subtracted amplitudes) observed in brain-behavior correlations that include words (regular/irregular) reading subtests, remains identical when words (regular/irregular) reading subtests are not included.

In grade 1, composite reading scores (average of single letters, syllables and pseudowords reading subtests) correlated with response amplitudes on O1 only (Spearman Rho=0.29; *P*=0.038) while in grade 2, composite reading scores correlated with response amplitudes on P7 only (Rho=0.50; p=0.001). No significant correlation was found with response amplitudes in the RH. However, after Bonferroni correction, only the correlation of composite reading scores with P7 in grade 2 remained significant (at an alpha of p=0.0124).

**Supplementary Analysis 3.** Replication of brain-behavior correlations between reading composite scores and discrimination responses of symbol strings (referring to point 2.3.2.)

The absence of relationship between composite reading scores and responses to symbol strings (baseline subtracted amplitudes) observed in brain-behavior correlations that include words (regular/irregular) reading subtests, remains observed when words (regular/irregular) reading subtests are not included.

Composite reading scores (average of single letters, syllables and pseudowords reading subtests) did not significantly correlate with response amplitudes to symbol strings in any grade (neither at an alpha of p=0.050 or p=0.0124, i.e., after Bonferroni correction). In grade 1, O1: Spearman Rho= -0.24; *P* =0.067, P7: Spearman Rho=0.04; *P*=0.399, O2: Spearman Rho=- 0.23; *P*=0.077, P8: Spearman Rho=0.005 ; *P*=0.489). In grade 2, O1: Spearman Rho=0.09;

*P*=0.300, P7: Spearman Rho=-0.07 ; *P*=0.342, O2: Spearman Rho=0.05; *P*=0.376, P8:

Spearman Rho=-0.09; *P*=0.291).

**Supplementary Table 1.** Percentage of response distributed across harmonics in grade 1 and grade 2, per electrode.

| **Grade 1** | | | | | |
| --- | --- | --- | --- | --- | --- |
| **Harmonic** | **LH** |  | **RH** |  | **Overall** |
|  | **O1** | **P7** | **O2** | **P8** |  |
| 1.2Hz | 41 | 42 | 39 | 41 | 41 |
| 2.4Hz | 31 | 29 | 31 | 30 | 30 |
| 3.6Hz | 20 | 22 | 22 | 20 | 21 |
| 4.8Hz | 8 | 6 | 9 | 10 | 8 |
| 7.2Hz | 0 | 1 | 0 | 0 | 0 |
|  | 100 | 100 | 100 | 100 |  |
| **Grade 2** | | | | | |
| **Harmonic** | **LH** |  | **RH** |  | **Overall** |
|  | **O1** | **P7** | **O2** | **P8** |  |
| 1.2Hz | 36 | 37 | 36 | 40 | 38 |
| 2.4Hz | 32 | 35 | 27 | 29 | 30 |
| 3.6Hz | 21 | 19 | 22 | 18 | 20 |
| 4.8Hz | 10 | 8 | 11 | 11 | 10 |
| 7.2Hz | 1 | 1 | 3 | 2 | 2 |
|  | 100 | 100 | 100 | 100 |  |
